# QUALITY OF LIFE USING EURAHS-QoL SCORES AFTER SURGICAL TREATMENT OF INGUINAL HERNIA: LAPAROSCOPIC TRANSABDOMINAL PREPERITONEAL (TAPP) AND LICHTENSTEIN TECHNIQUES

**DOI:** 10.1590/0102-672020240005e1798

**Published:** 2024-06-17

**Authors:** Rodrigo SANDERSON, Danilo Dallago DE-MARCHI, Jean Clever Bido CESÁRIO, Lucas Godoy Dias SANDERSON, Bruno ZILBERSTEIN

**Affiliations:** 1 São Leopoldo Mandic, Faculty of Medicine, Postgraduate Course – Campinas (SP), Brazil.

**Keywords:** General Surgery, Hernia, Inguinal, Minimally Invasive Surgical Procedures, Quality of Life, Cirurgia Geral, Hérnia Inguinal, Procedimentos Cirúrgicos Minimamente Invasivos, Qualidade de vida

## Abstract

**BACKGROUND:**

Results on quality of life after inguinal hernia surgery, such as esthetics, postoperative pain, period of absence from activities, and recurrence are a relevant topic since inguinal hernia affects 27% of men and 3% of women at some point in their lives, and should guide health policies to allocate resources more efficiently.

**AIMS:**

To evaluate the quality of life in the late postoperative period of inguinal herniorrhaphy regarding recurrence, pain, esthetics, and restriction in activities, comparing the minimally invasive techniques — the transabdominal preperitoneal (TAPP) and the conventional Lichtenstein.

**METHODS:**

A cross-sectional observational clinical study was conducted with the EuraHS-QoL questionnaire validated and translated into Portuguese, applied to patients after an average of 65 months postoperatively. Forty-five patients were assessed, 28 undergoing Lichtenstein and 17 undergoing TAPP. All were males aged between 18 and 87 years with a primary unilateral inguinal hernia. Recurrent or bilateral hernias, other concomitant abdominal wall hernias, patients who chose not to participate or who were not found, and female patients were excluded from the study.

**RESULTS:**

Regarding the domains pain, restriction, and esthetics, there was no difference between the two groups when examining quality of life. Neither group presented recurrence in the studied period.

**CONCLUSIONS:**

Both TAPP and Lichtenstein techniques presented similar results concerning quality of life when compared in the long-term.

## RESUMO

**RACIONAL:**

Os resultados de qualidade de vida após cirurgia de hérnia inguinal, como estética, dor pós-operatória, período de afastamento das atividades e recorrência é um tema relevante, uma vez que a hérnia inguinal atinge 27% dos homens e 3% das mulheres em algum momento da vida, e deveriam orientar as políticas de saúde para alocar recursos de forma mais eficiente.

**OBJETIVOS:**

Avaliar a qualidade de vida no pós-operatório tardio de herniorrafia inguinal quanto a recidiva, dor, estética e restrição de atividades, comparando as técnicas minimamente invasivas transabdominal pré-peritoneal (TAPP) e convencional, Lichtenstein.

**MÉTODOS:**

Estudo clínico observacional transversal com questionário *EuraHS-QoL* validado e traduzido para o português, aplicado em pacientes após média de 65 meses de pós-operatório. Foram estudados 45 pacientes, 28 submetidos a Lichtenstein e 17 submetidos a TAPP. Todos eram do sexo masculino, com idade entre 18 e 87 anos, com hérnia inguinal unilateral primária. Hérnias recorrentes ou bilaterais, outras hérnias concomitantes da parede abdominal, pacientes que optaram por não participar ou que não foram encontrados e pacientes do sexo feminino foram excluídos do estudo.

**RESULTADOS:**

Em relação aos domínios dor, restrição e cosméticos, não houve diferença entre os dois grupos na avaliação da qualidade de vida. Os dois grupos não apresentaram recidiva no período estudado.

**CONCLUSÕES:**

Tanto a técnica TAPP quanto a técnica de Lichtenstein, neste estudo, apresentaram resultados semelhantes, quando comparadas em longo prazo, no que diz respeito à qualidade de vida

## INTRODUCTION

The diagnosis of inguinal hernia is estabished through the patient’s history (anamnesis) and a physical examination that reveals bulging in the inguinal region, reducible after a Valsalva maneuver. The diagnosis is clinical, and clinical differentiation between direct or indirect hernia at the time of diagnosis is unnecessary, as it does not affect treatment^13,30^.

The preferred technique for surgical treatment of inguinal hernia, based on recent randomized clinical trials and meta-analyses, is the tension-free mesh repair^2^. This approach is consensus and is considered the “gold standard” for inguinal hernia repair by the American College of Surgeons^7,14^.

Hernias, in general, are considered preventable causes of death. According to some population studies, inguinal hernias are the most common type: approximately 25 out of every 100 men and 2 out of every 100 women have at least one inguinal hernia at some point in their lives^22,36^.

Once a hernia is detected, the approach is surgical. It is consensus that long-term quality of life is better in operated patients than in those for whom surgery is not performed, despite some cost-effectiveness studies suggesting that observation may be a cost-effective alternative in asymptomatic or minimally symptomatic male patients^33^. The hernia operation, for many authors, serves as an indicator of surgical quality provided by different institutions and countries, establishing a scientific basis for the critical evaluation of the procedure and the improvement of healthcare systems^3,11,21^.

Once mesh repair is chosen, the surgical approach becomes a significant point of discussion. Currently, both conventional and laparoscopic methods, with or without robotic assistance, are employed for the surgical repair of inguinal hernias, each with advantages and disadvantages. The surgeon’s view of the inguinal region’s anatomy may change depending on the chosen approach^16,17,23,35^.

In the conventional technique, after skin incision and dissection of subcutaneous tissue, the surgeon has the classic view of the inguinal canal with its surgical repairs (anterior superior iliac spine, pubic tubercle, inguinal ligament, conjoint tendon, spermatic cord and its contents surrounded by the cremaster muscle, internal and external inguinal rings, epigastric vessels, and ilioinguinal and iliohypogastric nerves)^27,39,40^.

The laparoscopic technique for inguinal hernia correction is regarded as a complex operation. It requires a longer learning curve due to the need for knowledge of the posterior anatomy of the inguinal region^33^.

Many studies have been published in recent years, reporting significant advantages of the minimally invasive laparoscopic or robotic approach over conventional repairs, such as reduced postoperative pain and complications, faster recovery, decreased chronic pain, and recurrence rates^4,5,38^. These findings increasingly encourage surgeons to seek training^10,15^. It is important to note that these techniques can be used in both elective and emergency procedures if clinical conditions permit^9,28^.

However, minimally invasive laparoscopic procedures require a longer learning curve, not to mention the implicit costs involved^31^. But would the benefits of this technique outweigh the costs?

Data from the Brazilian Hernia Society on June 7, 2019, revealed that 281,392 abdominal wall hernia surgeries were performed nationwide through the Unified Health System (SUS) between March 2018 and March 2019. Among these, only 1,745 (0.62%) were minimally invasive procedures^37^.

When analyzing the proportion of open and laparoscopic operations, there is a substantial gap between the techniques (99.4% vs. 0.6%, respectively). According to data from the Brazilian Unified Health System Department of Informatics (DATASUS), there was an increase in laparoscopic inguinal hernia surgeries from 0.28% to 0.41% between 2008 and 2018, representing a 46% growth. However, this value is relatively low compared to open procedures. These figures contrast with those in the United States, where laparoscopic techniques represent approximately 27% of procedures, and South Korea, where there was an increase from 2.4% to 29.5% in laparoscopic repairs between 2007 and 2015 in a population with full coverage by national health insurance^12^.

Currently, the issue of quality of life is a relevant topic in various fields of knowledge investigating economic, social, environmental, and territorial phenomena characterizing modern societies. This significance has sparked debates about theoretical developments related to quality of life^11,21^.

This understanding led the European Hernia Society to develop and publish the EuraHS-QoL in 2016, specifically designed for use in patients undergoing abdominal wall hernia surgery, as other tools for assessing the quality of life after hernia repair were not proven to be useful^20,26^.

The objective of this study was to analyze the characteristics of patients undergoing minimally invasive operations such as the laparoscopic TAPP and the conventional Lichtenstein techniques in primary unilateral inguinal hernia repair, as well as the comparison of the quality of life of these patients in the late postoperative period, at Santa Casa de Porto Ferreira, in a small city in the state of São Paulo (SP).

## METHODS

An observational cross-sectional clinical study was conducted on male patients undergoing unilateral laparoscopic and conventional inguinal hernia repair at Santa Casa de Misericórdia in Porto Ferreira, from January 2016 to December 2018. Data were collected from the medical records of 74 patients, being 47 operated on using the conventional Lichtenstein technique and 27 using the laparoscopic TAPP technique. Of the total, 29 patients could not be contacted as 25 did not respond and 4 had deceased.

Therefore, 45 patients were analyzed, of whom 28 were submitted to open inguinal hernia surgery using the Lichtenstein technique^23^, while 17 submitted to the fully stapled laparoscopic approach. In the latter, mesh fixation was performed with three to four non-absorbable staples, and peritoneal closure was completed with four non-absorbable staples. Most patients underwent elective surgery, except for two who underwent conventional surgery due to incarcerated hernias.

The study sample was non-probabilistic and was selected based on availability at the Digestive System Surgery Department of the above-mentioned institution. Surgeries were performed by a senior surgeon with at least 20 years of experience in laparoscopic surgery.

### Inclusion criteria

1. Adult male patients;
2. Aged 18 years or older and younger than 87 years;
3. Presence of primary unilateral inguinal hernia;
4. Patients who have signed informed consent form.

### Exclusion criteria

1. Recurrent or bilateral hernias;
2. Other concomitant abdominal wall hernias;
3. Female patients;
4. Patients who chose not to participate or could not be located.

### Study population

The study was conducted at Porto Ferreira Medical Center (private clinic) and Dr. Américo Montenegro Medical Specialties and Imaging Center (SUS outpatient clinic of the Municipal Government), in the city of Porto Ferreira (SP), where demographic data were collected from patients undergoing unilateral laparoscopic and conventional inguinal hernia repair, respectively.

The study underwent ethical review by the Medical Ethics Committee of the Municipal Government of Porto Ferreira and the Ethics Committee of the Porto Ferreira Medical Center, which was approved by both (No. 5,704,439).

### Quality of life questionnaire

**Figure 1:**
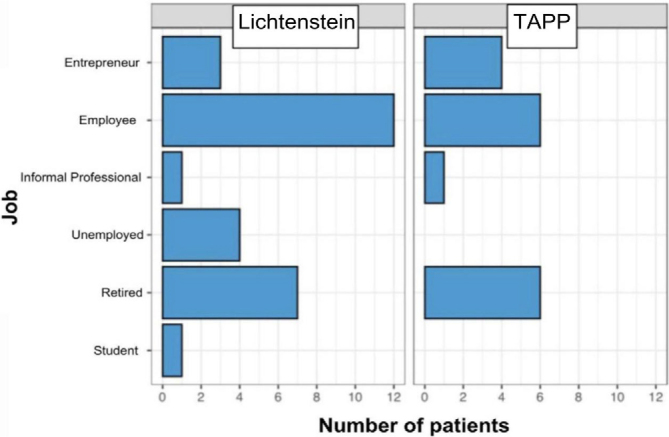
Work activities at the time of surgery.

Quality of life was assessed using the EuraHS-QoL, a specific questionnaire validated for abdominal wall hernia surgery, consisting of nine questions divided into three domains: pain, activity restriction, and esthetic discomfort. Already translated into Portuguese, this questionnaire was validated for inguinal hernia correction^26^. Each question is scored on an 11-point scale from 0 to 10, where 0 represents “absence of symptoms” and 10 represents “worst imaginable pain”, “completely restricted”, or “extremely ugly” for the three domains, respectively (Figure 1).

### Patient interviews

Patients were called and gathered at the local clinic Porto Ferreira Medical Center, where the research group administered the “Google Forms” questionnaire, a Google tool for creating online surveys based on standardized and validated questionnaires, including the EuraHS-QoL.

Patients were contacted by phone, and questions were answered spontaneously and in person, collected by two interviewers involved in the study. All participating patients signed informed consent form. The recorded data included demographic profile, pre-existing chronic diseases, postoperative complications, and postoperative quality of life. Data from all patients were used for descriptive statistics.

### Statistical analysis

A quantitative observational retrospective analysis was conducted on a cross-sectional observational clinical study. Based on the number of responses from a form, the study’s margin of error was calculated, using a significance level of 95%. The descriptive analysis aimed to present the distribution of collected observations. The inferential analysis included Fisher’s exact test, Shapiro-Wilk test, and Mann-Whitney U test.

## RESULTS

### Descriptive analysis of general population data

**Figure 2:**
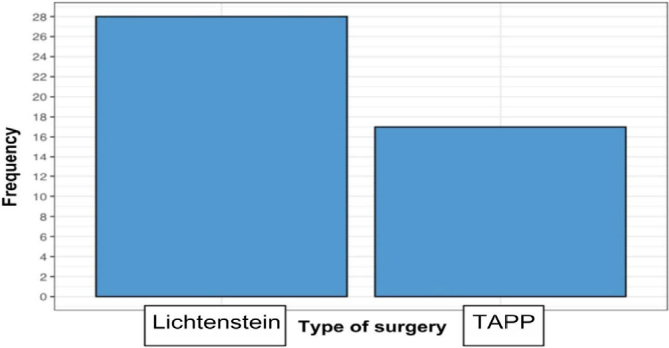
Surgical technique employed.

According to the graph below, out of the 45 observations, 28 patients underwent the Lichtenstein technique, and 17 underwent the TAPP (Figure 2).

**Figure 3:**
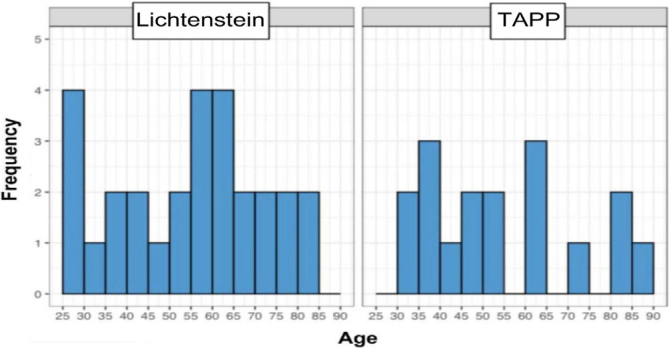
Age groups of patients undergoing two hernia correction techniques.

A histogram of age groups was created for each type of technique employed (Lichtenstein and TAPP), showing the frequency at the respective ages at the time of unilateral hernia surgery. Thus, it is possible to observe that among patients undergoing the Lichtenstein technique, the majority were in the age range of 25–30 years and 55–65 years, with a frequency of four individuals in the first age interval and eight in the second. On the other hand, the majority of patients undergoing the TAPP technique were in the age range of 35–40 years and 60–65 years, with three individuals in the first age group and two in the second (Figure 3).

Additionally, the boxplot of ages was also analyzed, which is a box diagram illustrating the interquartile range of the analyzed sample distribution, with the second quartile being the median of the distribution. Thus, it can be concluded that in surgery using the Lichtenstein technique, the youngest age was 27 years, the oldest age was 85 years, the first quartile was 41.25 years, the median was 57.50 years, the third quartile was at 66.50 years, and the mean age was 55.29 years. In the TAPP technique, the minimum age was 31 years, the maximum age was 87 years, the first quartile was 39.00 years, the median was 54.00 years, the third quartile corresponded to 64.00 years, and the mean age was 55.53 years.

**Figure 4:**
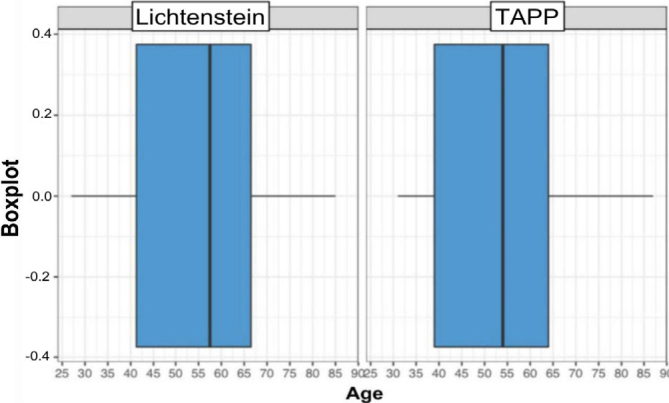
Boxplot of age groups.

It can be observed that the values of central measures (mean and median) for the two types of techniques employed have relatively close values. The boxplot allows for the observation of such similarities, even with different quantities of observations in each type of operation (Figure 4).

**Figure 5:**
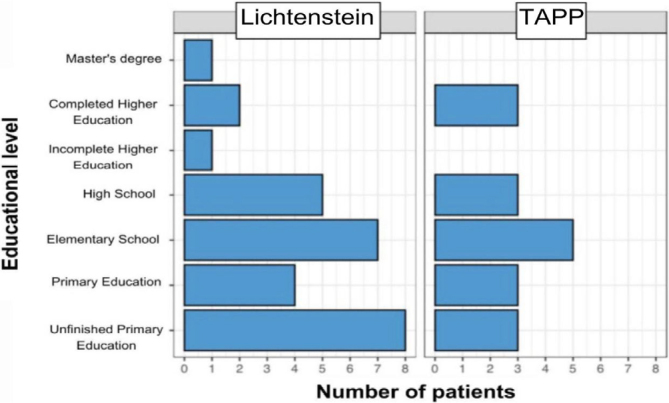
Education level of the studied population.

Another descriptive analysis was conducted regarding education. Figure 5 shows the education level of patients included in the study and the frequency of each level concerning both types of techniques employed. It is noted that the majority of patients submitted to the Lichtenstein technique had incomplete primary education, while most undergoing the TAPP had education up to elementary school. Thus, 30 patients out of the total 45 had completed elementary school, representing 66% of the sample (Figure 5).

When relating the occupational activity variable to each type of treatment, it can be concluded that approximately 43% of patients submitted to the Lichtenstein technique were employed. However, of patients undergoing the TAPP technique approximately 35% were employed and 35% were retired. Therefore, the conclusion was that 40% of patients subjected to inguinal hernia surgery were employed (Figure 1).

**Figure 6:**
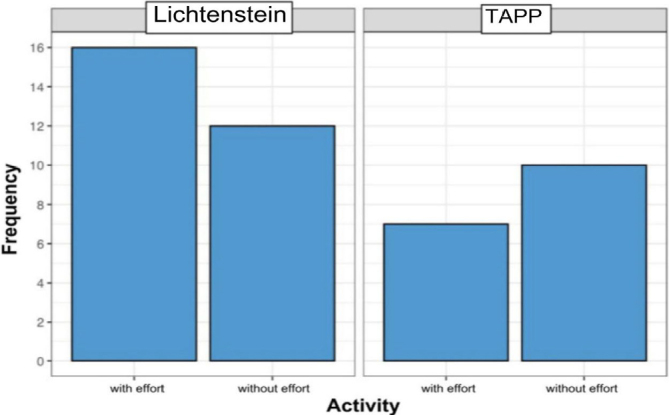
Type of activity at the time of surgery.

Additionally, the type of activity (with or without effort) performed by patients at the time of surgery was also analyzed. The majority of patients undergoing the Lichtenstein technique engaged in an activity with effort (approximately 57%). Conversely, the majority of patients undergoing laparoscopy performed an activity without effort (approximately 59%) (Figure 6).

**Figure 7:**
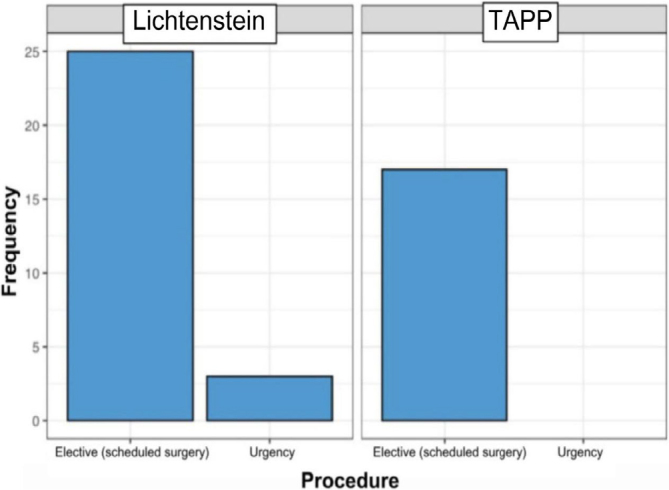
Priority of treatment.

When analyzing the type of procedure performed, it could be observed that 100% of patients who underwent surgery using the TAPP technique had planned the operation, compared to 89% of patients who underwent the Lichtenstein technique (Figure 7).

**Figure 8:**
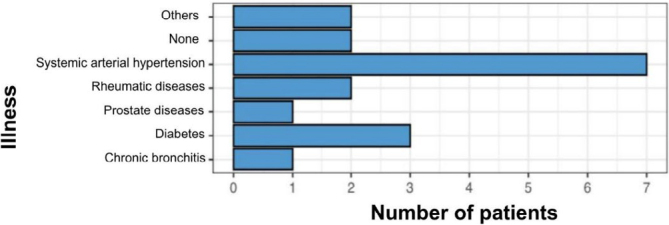
Comorbidities in the group undergoing the Lichtenstein technique.

**Figure 9:**
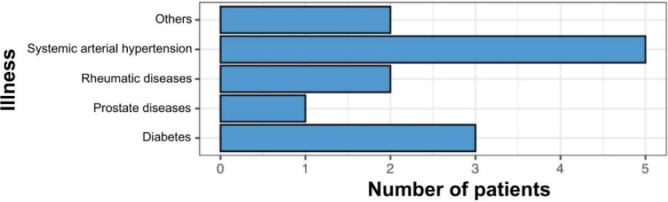
Comorbidities in the group undergoing the TAPP technique.

As for comorbidities at the time of surgery, it was evidenced that the majority had systemic arterial hypertension, accounting for 25% of patients submitted to the Lichtenstein technique and approximately 29% of those undergoing the TAPP technique (Figures 8 and 9).

### Descriptive analysis of the EuraHS-QoL questionnaire

**Figure 10:**
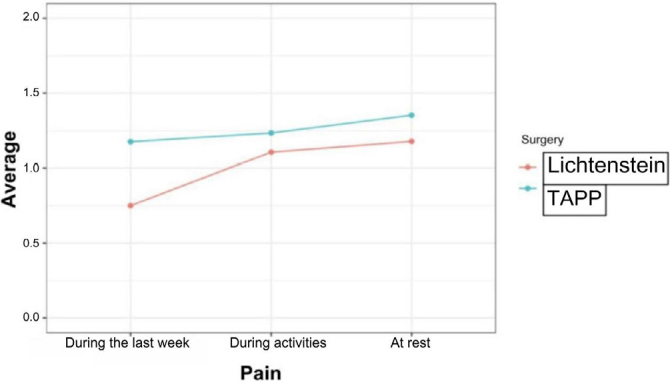
Average reported pain for each type of technique employed.

Now, using the EuraHS-QoL score^26^ to measure the results obtained from the two types of techniques employed, it was recognized that the average pain in surgery with the TAPP technique remained higher in all aspects. However, it is worth noting that the difference was not very significant as the Y-axis (axis of averages) is restricted between 0 and 2 (Figure 10).

**Figure 11:**
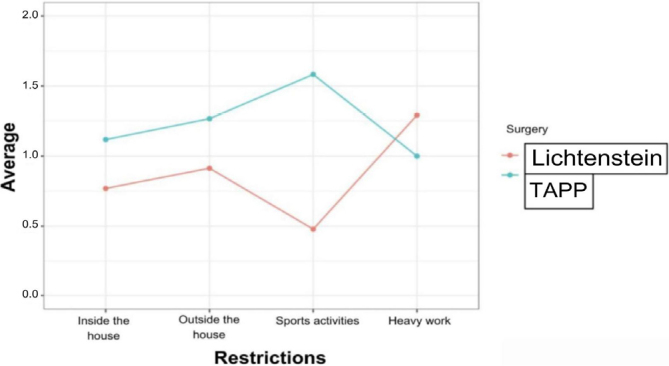
Average restrictions for each type of technique employed.

Similarly, surgery using the TAPP technique proved to be superior in almost all aspects of restriction, with inversion occurring only in relation to heavy work (Figure 11).

**Figure 12:**
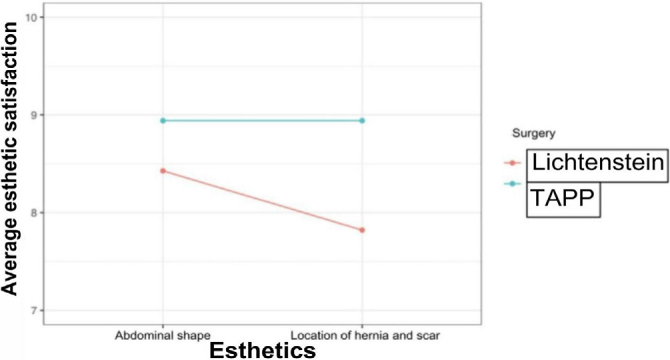
Average esthetic satisfaction for each type of surgery.

In the figure on the esthetics satisfaction of surgery, it can be noted that surgery using the TAPP technique showed a higher average in terms of aspects related to abdomen shape, hernia location, and scar compared to surgery using the Lichtenstein technique (Figure 12).

### Fisher’s exact test

The hypotheses were tested using the Fisher’s exact test to determine the presence or absence of dependence between categorical variables concerning the type of surgery. In the analyses, 2x2 contingency tables were created, always considering the surgical method used.

Upon analyzing the responses from the form, it was found that the complications that occurred were two swellings in surgeries using the Lichtenstein technique and one discharge of purulent secretion in surgery using the TAPP technique.

Initially, the hypothesis that the proportion of complications after surgery was higher in surgery using the TAPP technique than in surgery using the Lichtenstein technique was tested against the null hypothesis that there would be no difference in complications between the treatments.

Using the Fisher’s exact test, the p-value was 0.3163 (p>0.050). Thus, there was no evidence to assert that one technique had more complications than the other.

Regarding smoking, the relationship between patients who did or did not use cigarettes or similar products at the time of surgery and possible postoperative complications was analyzed. The alternative hypothesis was presented that there would be a relationship between the use of cigarettes or similar products at the time of surgery and postoperative complications. It is noteworthy that only three patients had postoperative complications in total, two with swelling in the TAPP group and one with purulent discharge in the Lichtenstein group. The p-value was approximately 0.3000 (p>0.050). Therefore, the evidence that smoking was associated with postoperative complications was not proven.

When evaluating whether operated patients should undergo surgery again and separating them by type of operation performed, the hypothesis was tested that the type of surgery would be related to the resubmission of the procedures against the null hypothesis that there would be no difference between them. In this test, the p-value was 0.6347 (p>0.050), which indicates that there was no difference between the groups regarding resubmission to surgery.

Finally, when analyzing whether the patient would recommend a procedure to a friend or family member, the hypothesis was tested that the type of technique would be related to the recommendation of the procedure against the null hypothesis that there would be no difference in recommendation between the treatments. In this test, the p-value was 0.3778 (p>0.050), which shows that there was no significant difference between the groups. Only one patient who had undergone TAPP surgery and reported an average equal to or greater than eight for variables related to pain after surgery stated that he would not recommend the technique.

### Shapiro-Wilk Test

The subsequent analyses employed the Shapiro-Wilk test to determine whether numerical variables (pain and restriction) follow a normal distribution or not. The null hypothesis that the numerical variables would follow a normal distribution was tested against the alternative hypothesis that the variables would not follow a normal distribution.

In this test, the p-values for all analyzed numerical variables were very close to zero. This implies that there was enough evidence to reject the null hypothesis that the variables would follow a normal distribution and confirms the alternative hypothesis that all the variables would not follow a normal distribution. As a result, it would be appropriate to use the Mann-Whitney U test, which is non-parametric.

### Mann-Whitney U Test

Mann-Whitney U test was used, as the Shapiro-Wilk test indicated that none of the variables followed a normal distribution.

Thus, the means and medians of the numerical variables were analyzed comparing the type of technique used.

Based on a possible association between the type of technique and the degree of pain during rest, the p-value was 0.8529 (p>0.050). Therefore, there was no evidence that one technique caused more postoperative pain than the other at rest (Table 1).

**Table 1:** Comparison of the means of postoperative quality of life between the Lichtenstein and TAPP techniques using the EuraHS Qol form.

	Lichtenstein n=28	TAPP n=17	p-value
Rest in pain	1.178571	1.352941	0.8529
Pain during activities	1.107143	1.235294	0.6218
Pain in the last week	0.750000	1.176471	0.6171
Restriction in physical activities	0.769231	1.117647	0.6151
Restrictions outside the home	0.913044	1.266667	1.0000
Restrictions during sport activities	0.478261	1.583333	0.1981
Restrictions during heavy work	1.291667	1.000000	0.5626
Shape of the abdomen	8.428571	8.941176	0.7249
Hernia site and scar	7.821429	8.941176	0.2783

TAPP: transabdominal preperitoneal.

Associating the type of technique used and the intensity of pain during activities, the p-value was 0.6218 (p>0.050). This means that there were no differences between Lichtenstein and TAPP operations regarding pain during activities (Table 1).

It was also not concluded that there was a significant difference between the operations when comparing the type of technique employed and the intensity of pain in the last week, and the p-value was 0.6171 (p>0.050) (Table 1).

In the domain restriction of daily activities, a possible association between the type of technique and the restrictions was tested. The p-value was 0.6151 (p>0.050), demonstrating no evidence that one operation restricts more than the other (Table 1).

Considering the relationship between the type of technique employed and restrictions outside the residence, the p-value was 1.0000 (p>0.050). In this case, the hypothesis states that there was no relationship between the type of operation and restrictions outside the domestic scope (Table 1).

For the domain restrictions during sports practice, seeking an association between the operations, the p-value was 0.1981 (p>0.050), which shows no significant difference between the operations (Table 1).

In the domain restrictions during heavy work, for a possible relation between the type of technique used and these restrictions, the p-value was 0.5626 (p>0.050). Therefore, the operations did not demonstrate a significant difference (Table 1).

When evaluating a possible correlation between the type of technique used and abdominal shape in the postoperative period, the p-value was 0.7249 (p>0.050). This result reveals that the operations did not show significant differences. In a possible association between the type of technique used and the scar’s shape, the p-value was 0.2783 (p>0.050) (Table 1).

## DISCUSSION

This cross-sectional observational clinical study provided information allowing for a comparison of the quality of life between TAPP and Lichtenstein surgeries. Its significant contribution lies in the handmade execution and the pioneering nature of its implementation in a small city with 55 thousand inhabitants without an established clinical research center. The major challenge, undoubtedly, was obtaining informed consent, which caused anxiety among patients and may explain the refusal of 25 individuals. Four deaths might be justified by the fact that data collection was carried out after the 2020/2021 pandemic.

Lichtenstein and TAPP techniques were comparable in various domains. They were primarily elective surgeries, with median ages of 57 and 54 years, respectively. The groups had similar comorbidities, predominantly hypertension and diabetes. However, the Lichtenstein group was engaged in more strenuous work activities and had a lower level of education^29^.

Currently, the main options for the surgical management of inguinal hernias are the conventional Lichtenstein technique and the TAPP laparoscopic approach^16,23,41^. However, there are significant differences in surgical conditions between wealthy and impoverished countries. The application of the technique takes into account the capabilities of the local healthcare system. The best technique is one that is easy, reproducible, comparable, with low recurrence rates, and amenable to statistical analysis^29^. Therefore, meta-analysis studies are currently the most effective in defining the best surgical method.

Scheuermann et al. conducted a multicenter randomized study with a meta-analysis of controlled studies comparing TAPP versus Lichtenstein^34^. The parameters included were surgical time, hospital stay, postoperative pain, complications such as seroma, hematoma, wall infection, return to work, and recurrence. The final analysis showed no significant differences between the two techniques from the perspective of the evaluated variables, similar to the results found in our research. In that study, however, TAPP demonstrated an advantage over Lichtenstein surgery, with less postoperative pain.

Postoperative complications and quality of life after surgery are considered the main and most important parameters in evaluating outcomes^6^. These findings are crucial when the surgical community reflects on current guidelines and their adoption in daily practice^20^. It is worth noting that in the UK, only 20% of primary unilateral inguinal hernias are repaired with laparoscopy, with significant variations between hospitals^25^. In Brazil, current data from the Brazilian Hernia Society demonstrate a gradual increase in the laparoscopic method.

The postoperative assessment of quality of life at only one moment, after an average of 65 months postoperatively, is an aspect that should be taken into consideration. The data collection method using the EuraHS-QoL questionnaire in this study was applied later, allowing for the measurement of differences in results between conventional and laparoscopic surgeries. The data collection time and the duration of follow-up vary widely among studies validating questionnaires, suggesting that the optimal follow-up time remains uncertain^18^. In this context, a literature review showed that quality of life does not significantly differ after 12 months of surgery^26^. The “pain in the last week” domain of the questionnaire does not apply in this study, although it does not show a statistical difference between the studied surgeries.

In contrast to other large studies on inguinal hernia, this research focused on primary unilateral inguinal hernias in male patients. Additionally, the analysis considered the laparoscopic TAPP group, as the service does not perform totally extraperitoneal (TEP) laparoscopic technique, even though evidence shows that these surgeries have similar risks of postoperative complications, such as chronic pain incidence and recurrence rates^8,33,35^. Male patients were chosen because, in the creation of the female peritoneal “flapping” in TAPP surgery, the round ligament is incised, which could contribute to bias in chronic pain, and the inguinal canal anatomy differs between men and women.

Large epidemiological studies from national databases show that reoperation rates after inguinotomy hernia repairs in women are higher than in men. In approximately 40% of reoperations after the use of an anterior mesh or non-mesh repairs, a recurrent femoral hernia is found^10^.

In the present study, conventional surgery had a single event with operative wound infection. In the TAPP group, two episodes of swelling (seroma and hematoma) were recorded. The statistical results of the research do not indicate association with tobacco. Additionally, the TAPP group presented higher pain in the descriptive analysis of the EuraHS-QoL questionnaire. However, it is worth noting that this was a very small difference, ranging 0–2, a factor that may be explained by the use of staples for peritoneal closure. Nevertheless, current studies show that techniques using staples, sutures, or tacks for peritoneal flap closure after TAPP do not present significant differences in operative outcomes, postoperative quality of life, or symptom resolution^32^. Some advocate not closing the peritoneal flap and using protected-face meshes without major repercussions^24^.

An extensive review by Aiolfi et al. in 2021 concluded that minimally invasive TAPP and TEP repairs seem to be associated with a significant reduction in early postoperative pain, return to work/activities, chronic pain, hematoma, and wound infection compared to tension-free Lichtenstein repair. Hernia recurrence, seroma, and hospitalization time appear similar between treatments^1^.

Typically, recurrence rates range between 1 and 4% with experienced surgeons worldwide^18^. In this study, the recurrence rate was zero for both Lichtenstein and TAPP techniques. These results are below those reported in the literature^19^, perhaps due to a short observation period.

Laparoscopic repair of inguinal hernia is a challenging operation for the surgeon, and there is evidence that it is associated with a learning curve where recurrence rates increase^19^. According to the European Hernia Society, to achieve results comparable to the ideal, at least 100 laparoscopic cases need to be performed. Additionally, the society states that complication rates decrease by 50% after completing 50 cases^26^.

## CONCLUSIONS

Significant differences in the quality of life, using the EuraHS-QoL questionnaire, were not recorded after primary unilateral inguinal hernia repair in male patients undergoing TAPP and Lichtenstein techniques when patients are analyzed in the late postoperative period. However, due to the small sample size, we should address the results with caution and avoid generalizing the conclusions.
